# Intrinsic Type 1 Interferon (IFN1) Profile of Uncultured Human Bone Marrow CD45^low^CD271^+^ Multipotential Stromal Cells (BM-MSCs): The Impact of Donor Age, Culture Expansion and IFNα and IFNβ Stimulation

**DOI:** 10.3390/biomedicines8070214

**Published:** 2020-07-15

**Authors:** Payal Ganguly, Agata N. Burska, Charlotte L.M. Davis, Jehan J. El-Jawhari, Peter V. Giannoudis, Elena A. Jones

**Affiliations:** 1 Leeds Institute of Rheumatic and Musculoskeletal Medicine, University of Leeds, Leeds LS9 7TF, UK; umpga@leeds.ac.uk (P.G.); A.N.Burska@leeds.ac.uk (A.N.B.); Charlottelmdavis@gmail.com (C.L.M.D.); p.giannoudis@leeds.ac.uk (P.V.G.); 2 Leeds Musculoskeletal Biomedical Research Centre, Chapel Allerton Hospital, Leeds LS7 4SA, UK; 3 Department of Biosciences, School of Science and Technology, Nottingham Trent University, Nottingham NG11 8NF, UK; jehan.el-jawhari@ntu.ac.uk; 4 Department of Clinical Pathology, Mansoura University, Mansoura 35516, Egypt

**Keywords:** aging, mesenchymal stromal cells, bone marrow, type 1 interferon, senescence

## Abstract

Skeletal aging is associated with reduced proliferative potential of bone marrow (BM) multipotential stromal cells (MSCs). Recent data suggest the involvement of type 1 interferon (IFN1) signalling in hematopoietic stem cell (HSC) senescence. Considering that BM-HSCs and BM-MSCs share the same BM niche, we investigated IFN1 expression profile in human BM-MSCs in relation to donor age, culture-expansion and IFN1 (α and β) stimulation. Fluorescence-activated cell sorting was used to purify uncultured BM-MSCs from younger (19–41, *n* = 6) and older (59–89, *n* = 6) donors based on the CD45^low^CD271^+^ phenotype, and hematopoietic-lineage cells (BM-HLCs, CD45^+^CD271^−^) were used as controls. Gene expression was analysed using integrated circuits arrays in sorted fractions as well as cultured/stimulated BM-MSCs and Y201/Y202 immortalised cell lines. IFN1 stimulation led to BM-MSC growth arrest and upregulation of many IFN1-stimulated genes (ISGs), with IFNβ demonstrating stronger effects. Uncultured MSCs were characterised by a moderate-level ISG expression similar to Y201 cells. Age-related changes in ISG expression were negligible in BM-MSCs compared to BM-HLCs, and intracellular reactive oxygen species (ROS) levels in BM-MSCs did not significantly correlate with donor age. Antiaging genes Klotho and SIRT6 correlated with more ISGs in BM-MSCs than in BM-HLCs. In patients with osteoarthritis (OA), BM-MSCs expressed considerably lower levels of several ISGs, indicating that their IFN1 signature is affected in a pathological condition. In summary, BM-MSCs possess homeostatic IFN1 gene expression signature in health, which is sensitive to in vitro culture and external IFN1 stimulation. IFN signalling may facilitate in vivo BM-MSC responses to DNA damage and combating senescence and aberrant immune activation.

## 1. Introduction

Aging-associated health and well-being concerns have increased in the last two to three decades [1] owing to the global demographic shift towards people aged over 60 [2]. While several hallmarks of aging have been suggested as the underlying causes of age-related conditions [3], the exact mechanisms leading to deterioration of health at advanced age, including decline in immunity and increased frailty are yet to be established.

Among the age-related diseases, those associated with the skeletal system are the leading contributors of disability worldwide according to the World Health Organisation [4]. The skeletal system forms the basic framework of the body, protecting internal organs and performing movements and other mechanical and homeostatic functions. Certain bones also contain active bone marrow (BM), which is the site of haematopoiesis, where various cell types are formed owing to the activity of BM-resident hematopoietic stem cells (BM-HSCs) and multipotential stromal cells (BM-MSCs) [5]. With advancing age, the essential activities of both BM-HSCs and BM-MSCs appear to decline, resulting in a phenomenon known as inflamm-aging and age-related bone loss [6]. Inflamm-aging conceptually postulates an intrinsic decline of the proliferation potential and increase in senescence and DNA damage in immune-lineage cells. This is accompanied by the reduction of protective extrinsic mechanisms within the cellular microenvironment that aim at removal of senescent cells and accumulated damage, that is, autophagy [7]. Among the most noted hallmarks of inflamm-aging is the preferential increase in the myeloid lineage cell production, also known as “myeloid skewing” [8]. Age-related bone loss, on the other hand, is associated with inefficient bone remodelling processes [9] and cell senescence, mainly due to oxidative stress and DNA damage, as well as telomere erosion [10,11].

Considering that BM-MSCs locally participate in multiple cellular processes, including bone formation and the control of bone remodelling, as well as BM-HSC support activity [12], understanding age-related changes in BM-MSCs is of considerable importance. Recently, several studies have documented an age-related decline in the number and particularly, colony sizes of human BM-MSCs, identified using CD271 or SSEA4 marker expression [13,14,15]. Within these phenotypes, multipotential BM-MSCs may be replaced with dysfunctional and presenescent stromal cells in aged individuals. However, this process has not yet been documented formally in humans owing to the low frequencies of BM-MSCs, as well as difficulties of in situ identification of senescent cells in bone [11].

In BM-HSCs, cell senescence triggered by oxidative stress and DNA damage is mediated via the cytosolic DNA-sensing cGAS/STING pathway, which leads to an activation of cell-intrinsic type-1 interferon (IFN1) response [16,17]. Type-1 interferons (IFN1) bind to their receptor subunits IFNα 1 and 2 (IFNAR1 and IFNAR2) and signal using the Janus kinase and signal transducer and activation of transcription (JAK-STAT) pathway. Initially described as innate immune cells’ response to viral pathogens (extrinsic IFN1 response), homeostatic IFN1-stimulated gene (ISG) upregulation in other cells, including BM-HSCs and other types of stem cells, have been more recently connected to a cell’s control of autophagy, proliferation, apoptosis, and senescence (intrinsic IFN1 response) [16,18,19]. Interestingly, distinct IFN1 profiles of in-vitro-expanded BM-MSC clones have been recently linked to MSC subpopulations with different proliferation, migration and immunomodulation capacities [20]. Furthermore, BM-MSC senescence in patients with systemic lupus erythematosus (SLE), an autoimmune disease in which IFN1 is known to be produced in excess [21], has been found to be associated with an upregulation of certain IFN1 ISGs [20,22,23]. Collectively, these data indicate a possibility of age- and disease-related changes in IFN1 gene expression profiles of BM-MSCs; however, very little is yet known about IFN1 expression signature in BM-MSCs in vivo or following standard expansion or IFN1 stimulation.

Based on the fact that BM-MSC gene expression levels change significantly after in vitro expansion [24,25], the aim of this study was to investigate intrinsic IFN1 gene expression profile of human BM-MSCs and to explore any potential changes in this profile in relation to donor age, culture-expansion or IFN1 stimulation. MSCs were purified using the commonly-accepted CD45^low^CD271^+^ phenotype [13,15] and their IFN1 gene expression was compared to donor matched hematopoietic-lineage cells (BM-HLCs: CD45^+^CD271^−^) as well as cultured and interferon α- and β-stimulated BM-MSCs. It was hypothesised that (1) BM-MSCs would be responding to IFN1 stimulation, (2) IFN1 gene expression would be different between uncultured and cultured BM-MSCs, (3) the expression of certain ISGs could change in older donors MSCs due to increased DNA damage and (4) that IFN1 profiles of BM-MSCs would be different to BM-HLCs, reflecting their different homeostatic functions within the BM. We also hypothesised that BM-MSC and BM-HLC IFN1 gene expression profiles could be altered in osteoarthritis (OA), a disease characterised by aberrant local and systemic immune responses [26,27].

## 2. Materials and Methods

### 2.1. Patients and Cells

Bone marrow aspirate (BMA) was collected from 15 donors undergoing fracture correction or removal of metal work that were otherwise healthy. Cells from 12 donors (*n* = 6 young, range 19–40 years old and *n* = 6 old, range 59–89 years old) based on age groups boundaries defined earlier [15] were used for cell sorting and native BM-MSC IFN1 profile evaluation. Cells from three donors (aged 23, 49 and 59 years old) were used for IFN1 stimulation experiments. For the OA part of the study, BM-MSCs were extracted from femoral heads of seven patients (age range 56–82 years old), as previously described [28,29], and their BM-MSC IFN1 gene expression profiles were compared with older healthy donors (*n* = 6, range 59–89 years old). The ethical approval for the study was provided by the Yorkshire and Humberside National Research Ethics Committee (reference 06/Q1206/127, 11 April 2012) and the Yorkshire & The Humber—South Yorkshire Research Ethics Committee (reference 14/YH/0087, 17 July 2014). Written informed consent was obtained from all patients.

### 2.2. Sample Processing and Cell Sorting

An average of 8 mL of BMA was collected in ethylenediaminetetraacetic acid (EDTA) tubes and filtered through 70 μm cell strainer (Thermo Fisher Scientific, Warrington, UK)) to remove any cellular clumps. Next, the red blood cells were lysed using 1:9 BMA: ammonium chloride (*v*/*v*) by incubation at 37 °C for 5 min. The BMA and ammonium chloride mix was then centrifuged at 300× *g* for 10 min at room temperature (RT) and the obtained cell pellet was further washed twice with phosphate buffer saline (PBS) (Thermo Fisher Scientific) to eliminate traces of remaining ammonium chloride. Following counting, the cell suspension was centrifuged at 300× *g* for 10 min at RT and frozen in DMEM with 45% foetal bovine serum (FBS) and 10% dimethylsulfoxide (DMSO). The frozen samples were defrosted, washed with PBS to remove any traces of DMSO and then enriched with anti-fibroblast magnetic beads (Miltenyi Biotec, Bergisch Gladbach, Germany) as previously described [24] and then subjected to cell sorting to separate BM-MSCs (CD45^low^CD271^+^) and BM-HLCs (CD45^high^CD271^−^) cells, as previously described [15]. In brief, enriched cells were stained with the following antibodies for 15 min: 20 μL CD45 V450 and 20 μL CD271 PE Vio770 (both from BD Pharmingen, Oxford, UK) and 10 μL of 7-AAD (BD Pharmigen) was added to the cells immediately prior to cell sorting, which was performed using a BD Influx cell sorter [15]. For each antibody, isotype controls were used to control for nonspecific antibody binding. Sorted cells of the following populations CD45^low^CD271^+^ (BM-MSCs) and CD45^high^CD271^−^ (BM-HLCs) were collected into 1.5 mL Eppendorf tubes containing 350 μL guanidinium-based lysis buffer (Buffer RL, Norgen Biotek, Thorold, Canada), and lysates were frozen at −80 °C for subsequent RNA extraction and qPCR. The number of BM-MSCs collected after sorting was in the range of 647 to 22,2229 cells (median of 4694 cells), whereas the number of BM-HLCs collected was always 70,000 cells The expression of 102 genes (Supplementary Table S1) was investigated in both the populations. Multipotential BM-MSC genes were investigated for potential changes in differentiation and proliferation in Flex six^TM^ IFC, whereas, IFN1 genes were investigated in 48.48.gene IFC^TM^ (both Fluidigm, San Francisco, California, USA).

### 2.3. Quantitative Polymerase Chain Reaction (qPCR)

RNA was extracted from cell lysates obtained from cell sorting described in [15] using Single Cell RNA Purification Kit (Norgen Biotek) following manufacturer instructions with genomic DNA removal using DNAse I from the same company. The eluted RNA was reverse transcribed, preamplified for 18 cycles using Fluidigm reagents as per the outlined manufacturer protocols. Quantitative PCR (qPCR) was then performed using 48.48 Dynamic Array™ IFC on BioMark HD. The Ct values for genes of interest were normalised to the endogenous control HPRT-1 using the formula ΔCt = Ct_target gene_ − Ct_housekeeping gene_ and relative expression was calculated as 2^−ΔCt^ and used for statistical analysis. The Cluster and Treeview softwares were used to generate clusters of gene expression data for visualisation of heatmaps across donor cells and stimulations. The clusters are presented by the level of expression of genes, highest indicated in red (+ 3) to lowest indicated in green (−3). The grey squares indicate data points that were below detection level.

### 2.4. BM-MSC Culture and IFN1 Stimulation

Y201 and Y202 immortalised BM-MSC cell lines [20] as well as primary BM-MSCs from unsorted BM were cultured in StemMacs MSC expansion media (Miltenyi Biotec) containing 1% (*v*/*v*) penicillin streptomycin (PS) for three passages prior to stimulation or gene expression study, as previously described. Twenty-four hours prior to IFN1 stimulation, cells were seeded in duplicate wells of six-well plates at a density of 25,000 cells per well in 1 mL of expansion media. The following day, media was removed and cells were stimulated with 2 mL expansion media containing varying concentrations of IFNα or β (STEMCELL technologies Vancouver, Canada and Pepro Tech, London, UK, respectively) for up to 3 days (Supplementary Figure S1). Based on BST2 gene expression, 10 ng/mL concentration was selected for subsequent experiments and cells were cultured for up to 7 days. Control wells containing only expansion media were prepared in duplicate for all time points. Cells to be harvested on day 7 underwent a half media change with the same media on day 3. Cells were harvested on days 3 and 7 days post stimulation to assess both proliferation and gene expression. Gene panel used for this part of the study is shown in Supplementary Table S2.

On the day of harvesting, stimulation media was removed, wells were washed with 1 mL Dulbecco’s Phosphate Buffered Saline (DPBS) twice and cells harvested using Trypsin/EDTA (both from Sigma, Poole, UK). The effects of IFNα or β on BM-MSC proliferation were assessed via cell counting and confirmed, in selected experiments, by DNA quantification. After counting, all cells were lysed for RNA extraction and gene expression evaluation, as explained in the section above.

### 2.5. Intracellular ROS Measurements

BMA samples from young (*n* = 7) and old donors (*n* = 6) were treated with ammonium chloride to attain red cell lysis and washed with PBS. A total of 2 × 10^6^ cells were added to two flow cytometry tubes. The cells in the first tube were left as it is (to measure basal ROS) and the second tube was treated with tertbutyl hydrogen peroxide (Thermo Fisher Scientific) to generate induced ROS, as described previously [30], and was used as a positive control for the assay. Both the tubes were placed in the incubator for an hour, washed with PBS and then stained with 20 μL CD45 V450, 20 μL CD 271 PE-Vio770, and 2 μL CellROX FITC (Thermo Fisher Scientific) for 15 min and washed. CellROX is a nonfluorescent dye under reduced state, which upon oxidation, exhibits fluorescence. After incubation, the cells were washed in PBS, the supernatant was discarded and the cells were resuspended in 100 μL of medium (DMEM + 10%FBS). Finally, 2 μL SYTOX APC (Thermo Fisher Scientific) was added to distinguish between live and dead cells for 15 min. The cells were then analysed for the production of intracellular ROS using flow cytometry (LSRII, BD Pharmigen) following gating on live CD45^low^CD271^+^ BM-MSCs, and the data was analysed using FACS Diva version 8 (BD Pharmigen).

### 2.6. Statistics

Statistical analysis and graphics were performed using GraphPad Prism software (version 7.0a, San Diego, California, US). The normal distribution of the data was assessed using the Shapiro–Wilk and Kolmogorov–Smirnov tests for normality. For data found not to be normally distributed, the Mann–Whitney test was used for unpaired data and Wilcoxon-signed rank test for paired data. Statistical analysis of BM-MSC stimulation data across three time points (day 0/day 3/day 7) was carried out using one-way ANOVA followed by Kruskal–Wallis nonparametric test with Dunn correction for multiple groups. The results were considered significant at *p* value of <0.05.

## 3. Results

### 3.1. IFN1 Profile and Reduced Proliferation of Cultured BM-MSCs upon IFN1 Stimulation

**Figure 1:**
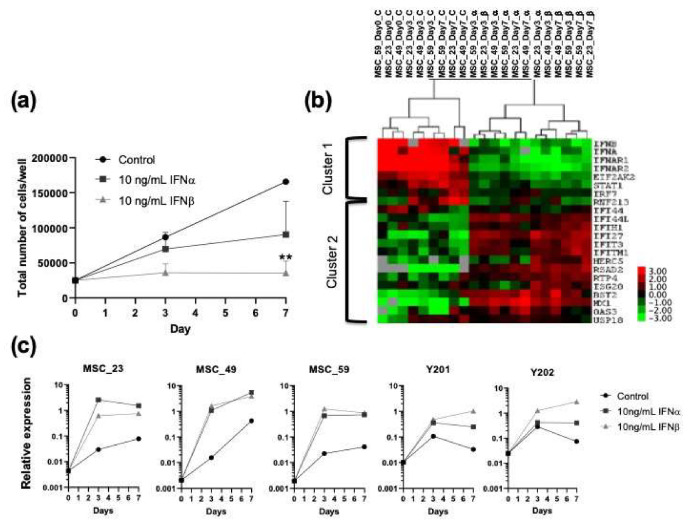
Growth and IFN1 profile changes in cultured bone marrow multipotential stromal cells (BM-MSCs) following IFNα or β stimulation. (**a**) Reduced proliferation in BM-MSC cultures upon exposure to IFNα or β. Three-way analysis of variance (ANOVA) followed by Kruskal–Wallis test, ** *p* < 0.01, *n* = 3 donors, symbols represent means, error bars indicate ± standard error of the mean (SEM). (**b**) IFN1 profile in BM-MSCs without and with IFNα or β stimulation. Cluster analysis of BM-MSC gene expression (*n* = 3 donors, label numbers indicate donor ages). Dendograms on the top indicate samples’ clustering: the symbols “α” and “β” indicate treatment with IFNα and IFNβ, respectively, and “c” indicates control nonstimulated samples. Cluster 1 and 2 indicate transcripts down- and upregulated following IFN1 stimulation, respectively. The colour key on the right indicates the range of expression levels with highest expression red and lowest in green. Grey squares indicate data points that were below detection. (**c**) Time-course expression of a representative Cluster 2 transcript USP18 on BM-MSCs from three individual donors and control cell lines Y201 and Y202.

Different IFN1 profiles with upregulation of certain ISGs have been previously linked to stem cell quiescence, senescence and proliferation [18,23,31]. To address our first aim and explore any differences in IFN1 gene expression profile between unstimulated and stimulated BM-MSCs, primary cultured BM-MSCs were treated with 10 ng/mL IFNα or IFNβ and their growth kinetics and IFN1 profiles were compared (Figure 1). Reduction in BM-MSC proliferation was observed following stimulation with either IFNα or IFNβ, consistent with early reports [32,33], and was statistically significant for IFNβ on day 7. In the absence of IFN1 treatment, BM-MSCs continued to proliferate and increased in numbers 3.4- and 6.6-fold on days 3 and 7, compared to day 0, respectively. The inhibitory effect of IFNβ was more pronounced than IFNα and induced a complete growth arrest of BM-MSCs (Figure 1a). BM-MSCs from the oldest donor (59 years old) displayed the slowest proliferation rate and were more inhibited by IFNα (stopped proliferating on day 3) compared to younger donors (23 and 49 years old). Similar to primary BM-MSCs, clonal cell lines Y201 and Y202 responded well to IFN1 stimulation and, similarly, their growth was more inhibited by IFNβ than by IFNα (Supplementary Figure S1).

Cluster analysis of topical IFN1 transcripts revealed the presence of two clusters (clusters 1 and 2) of genes (Figure 1b). Cluster 1 included transcripts for type I interferons (IFNα and IFNβ), their receptors IFNAR1 and IFNAR2, and the intermediate signalling molecule STAT1 [34]. These genes were expressed at a higher level in unstimulated, proliferating BM-MSCs compared in IFNα or IFNβ stimulated BM-MSCs, regardless of the day of culture. This suggested a suppression of IFN signalling and autocrine IFN production in the presence of external IFNs. Cluster 2 contained large numbers of ISGs, including BST2 and Mx1, that were previously shown to be expressed in native and cultured BM-MSCs (Figure 1b) [20,35]. Interestingly, within the stimulated samples, those treated with IFNβ showed a more distinct separation from unstimulated BM-MSCs, compared to BM-MSCs stimulated with IFNα, suggesting that gene expression of a majority selected ISGs was more strongly induced by IFNβ than IFNα (i.e., HERC5 and ISG20) (Figure 1b). Time-course expression of a representative Cluster 2 transcript USP18 on BM-MSCs from three individual donors, as well as control cell lines Y201 and Y202, is shown on Figure 1c. These data revealed clear differences in IFN1 gene expression profiles between cultured, proliferating BM-MSCs and IFN1-stimulated, growth inhibited BM-MSCs. Based on gene clustering, donor age appeared to have a lesser effect on ISG expression in BM-MSCs compared to IFN1 type or treatment duration (3 or 7 days).

### 3.2. IFN1 Profile of Uncultured CD45^low^CD271^+^ BM-MSCs in Comparison to Cultured and IFN1 Stimulated BM-MSCs

**Figure 2:**
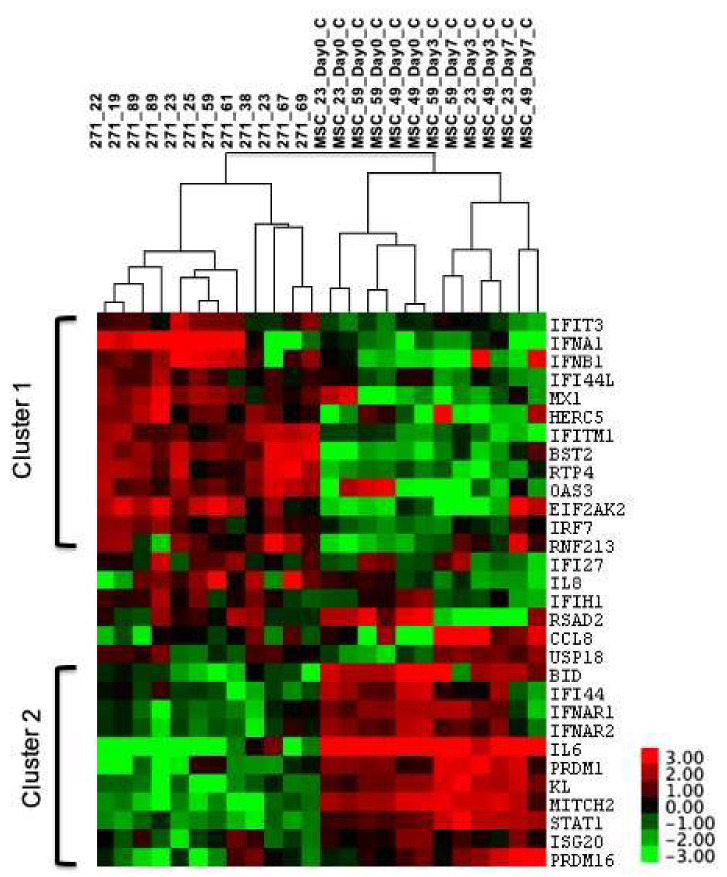
Cluster analysis of uncultured and cultured BM-MSCs. Uncultured BM-MSCs were purified based on the CD45^low^CD271^+^ phenotype (*n* = 12 donors) and cultured BM-MSCs were harvested from control wells with no stimulations on days 0, 3 and 7 of culture (*n* = 3 donors, label numbers indicate donor ages). Dendrograms on the top indicate the samples: CD271+—uncultured BM-MSCs, MSC—cultured BM-MSCs, day 0 BM-MSC samples were performed in duplicate. Clusters 1 and 2 indicate transcripts down- and upregulated following culture, respectively. The colour key on the right indicates the range of expression levels with highest expression red and lowest in green. Grey squares indicate data points that were below detection.

**Figure 3:**
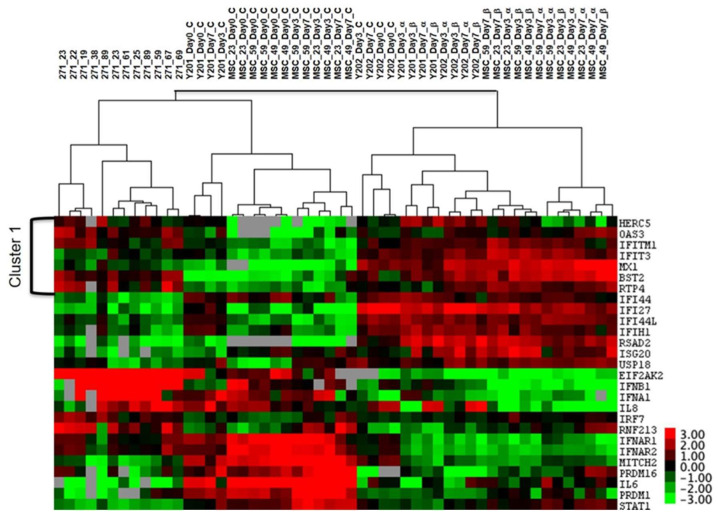
Cluster analysis of uncultured, cultured and IFNα- or IFNβ-stimulated BM-MSCs and BM-MSC cell lines Y201 and Y202 without or with IFNα or β stimulation. Uncultured BM-MSCs were purified based on the CD45^low^CD271^+^ phenotype (*n* = 12 donors), and cultured BM-MSCs were harvested from control wells with no stimulation on days 0, 3 and 7 of culture (*n* = 3 donors, label numbers indicate donor ages). Dendograms on the top indicate the samples: CD271^+^—uncultured BM-MSCs, MSC—cultured BM-MSCs, Y201/Y202—cell lines, α—stimulated with IFNα, β—stimulated with IFNβ. Cluster 1 indicates transcripts expressed at similar levels in uncultured and IFN1-stimulated BM-MSCs. The colour key on the right indicates the range of expression levels with highest expression red and lowest in green. Grey squares indicate data points that were below detection.

As documented in previous independent studies, standard BM-MSC culture expansion leads to downregulation in the expression of a large number of genes, including ISGs [24,25,36]. To get insights into the in vivo IFN1 “status” of uncultured BM-MSCs, the IFN1 profile of purified CD45^low^CD271^+^ cells was first compared to standard cultured BM-MSCs (nonstimulated, Figure 2). The analysis subsequently included BM-MSCs cultured in the presence of IFNα or IFNβ, as well as immortalised BM-MSCs cell lines Y201 (differentiation competent) and Y202 (immunomodulatory) previously shown to radically differ in their IFN1 and ISG profiles [20] (Figure 3).

Cluster analysis of the 30 selected genes in purified and culture expanded BM-MSCs revealed distinct segregation of purified native BM-MSCs (labelled as CD271^+^) from the cultured BM-MSCs, whereas the effects of donor age were less apparent (Figure 2). The expression of interferons (IFNα and IFNβ), as well as several well-known ISGs, including Mx1, HERC5, IFITM1, BST2, and IRF7, were distinctly downregulated following culture (Cluster 1, Figure 2). In contrast, another distinct cluster contained genes that were upregulated in culture, included IFN receptors genes (IFNAR1 and IFNAR2), STAT1, ISG20, as well as BID and MITCH that connect DNA-damage-associated IFN1 signalling with reactive oxygen species (ROS) accumulation and cell senescence [37]. Higher levels of autocrine IFN1 transcription and lower levels of IFN receptors’ transcription in uncultured BM-MSCs suggested that the in vivo BM-MSC niche may be relatively rich in IFN1 compared to in vitro conditions.

A broader view, with the inclusion of IFNα or IFNβ stimulated BM-MSCs as well as Y201 and Y202 BM-MSC cell lines, revealed further interesting findings. Firstly, uncultured/purified BM-MSCs and cultured BM-MSCs were clearly segregated from IFN1-stimulated BM-MSCs and were more similar to each other than to stimulated BM-MSCs (Figure 3). Secondly, Y201 data clustered nearer to cultured BM-MSCs, while most Y202 data clustered clearly with stimulated BM-MSCs. This was reassuring as Y202 cells were originally described as clonally expanded BM-MSCs with a more prominent IFN1 profile than Y201 cells [20]. Thirdly, a cluster of genes was identified that had similar levels of expression in uncultured and stimulated BM-MSCs. The genes within this cluster included OAS3 and BST2 previously described as associated with human BM-MSCs [20,35,38], as well as IFITM3 and RTP4 (Cluster 1, Figure 3).

### 3.3. Intrinsic IFN1 Profile of Uncultured CD45^low^CD271^+^ BM-MSCs in Comparison to Control CD45^+^CD271^−^ BM-HLCs

Cell type specificity in IFN1 profiles have been previously reported for immune-lineage cells [39] and different types of stem cells [18]. To investigate if IFN1 profile specificity exists in different-lineage cells inhabiting the same BM environment, IFN1 gene expression in CD45^low^CD271^+^ BM-MSCs was next compared to CD45^+^CD271^−^ BM-HLCs. Given only minor age-related differences observed so far, the comparison was first performed for the whole donor cohort (*n* = 12, age range 19–89 years old) and additional genes included molecules linked to the IFN1 > BID > ROS pathway, connecting IFN1 signalling and cellular senescence (BID and MTCH2) [17,40], two topical antiaging genes (KL and SIRT6) [41] and two genes indicating senescence-activated secretory phenotype (SASP) (IL6 and IL8) [31].

**Figure 4:**
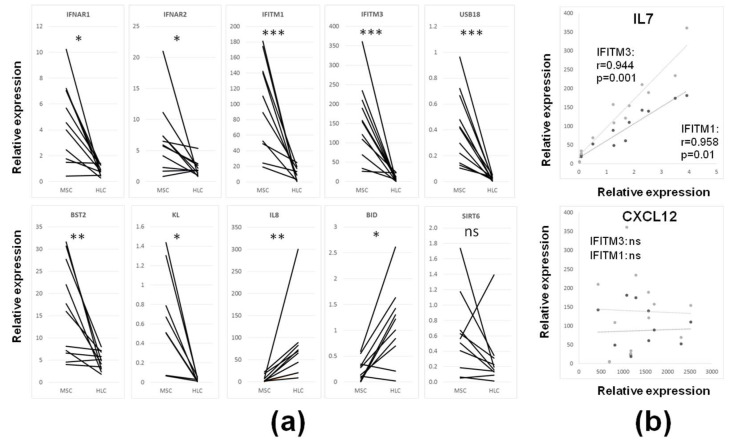
Expression of selected ISGs in uncultured BM-MSCs (CD45^low^CD271^+^ cells) and control BM-HLCs (CD45^+^CD271^−^ cells). (**a**) Expression of genes that were higher expressed in BM-MSCs (first seven panels) or BM-HLCs (last three panels). Wilcoxon-signed rank test for matched paired data, * *p* < 0.05, ** *p* < 0.01 and *** *p* < 0.001, ns—nonsignificant. (**b**) Correlation of the expression of BM stroma-producing cytokines IL7 and CXCL12 with the expression of IFITM1 and IFITM3 in uncultured BM-MSCs. Spearman rank correlation test, IFITM1—dark symbols, IFITM3—light symbols.

Despite the fact that the majority of ISGs were previously shown to be expressed in immune cells [35], and were therefore expected to have increased expression in BM-HLCs, the opposite was found here, whereby a larger number of ISGs displayed significantly higher expression in BM-MSCs (Table 1). For example, BM-MSCs expressed higher levels of transcripts for both IFN1 type receptors IFNAR1 and IFNAR2, transmembrane antiviral effectors IFITM1 and IFITM3, recently suggested to be important in stem cell differentiation [18], as well as BST2, USP18, and KL (Figure 4a).

As IFN1 signalling may be directly implicated in lineage-commitment or trophic functions of BM-MSCs [18], the relationships between the expression of key BM-MSC lineage transcripts (FABP4, PPARγ for adipogenesis, RUNX2, SPARC for osteogenesis and SOX9, ACN for chondrogenesis) were next correlated with their expression of IFN1 receptors or ISGs and no significant correlations were found (data not shown). Contrastingly, the expression of IL7, a well-known BM stromal cytokine involved in lymphoid progenitor development and B and T memory cells retention in the BM [42,43] strongly positively correlated with many ISGs including highly-expressed IFITM1 and IFITM3 (Figure 4b). However, the latter ISGs did not correlate with the transcript levels for another BM-MSC stromal-support molecule CXCL12, which is involved in the BM-MSC support for the maintenance of BM-HSCs [12,44] (Figure 4b). Interestingly, the expression of TNFSF11 (RANKL), a molecule promoting bone resorption [45], had significant negative correlation with four other ISGs: Ly6E (*r* = −0.7692, *p* = 0.005), LRP1 (*p* = −0.7273, *p* = 0.01), BST2 (*r* = −0.762, *p* = 0.01) and OAS3 (*r* = −0.8252, *p* = 0.002).

Conversely, BM-HLCs were characterised by higher-level expression of IL8, as well as BID, a proapoptotic molecule that regulates mitochondrial ROS in the DDR-induced senescence induction pathway in BM-HSCs (Table 1 and Figure 4a) [46]. The expression of SIRT6 was higher in BM-MSCs in most donors, but the differences failed to reach statistical significance (Figure 4a). These data revealed distinct differences in IFN1 profiles in stromal and hematopoietic-lineage cells inhabiting the same BM environment.

### 3.4. Age-Related Changes in IFN1 Profile of CD45^low^CD271^+^ BM-MSCs Compared to CD45^+^CD271^−^ BM-HLCs

**Figure 5:**
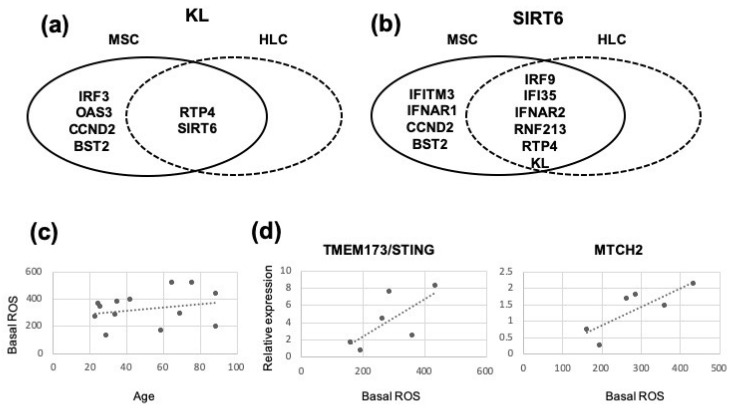
Venn diagrams showing expression of antiaging genes in correlation with ISGs in uncultured BM-MSCs (CD45^low^CD271^+^ cells) and control BM-HLCs (CD45^+^CD271^−^ cells) and reactive oxygen species (ROS) production in uncultured BM-MSCs in relationship to donor age and TMEM173/STING and MTCH2 expression levels. (**a**) ISGs significantly correlating with KL and (**b**) ISGs significantly correlating with Sirt6, in BM-MSCs, BM-HLCs and both types of cells. (**c**) Basal intracellular ROS levels in uncultured BM-MSCs (CD45^low^CD271^+^ cells) from 13 healthy donors. (**d**) Positive relationships between basal ROS levels in uncultured BM-MSCs (CD45^low^CD271^+^ cells) and their expression of TMEM173/STING (left) and MTCH2 (right).

Compared to younger donors’ BM-HLCs (19–40 years old), a number of genes displayed statistically significant, over 2-fold reduction in older donors’ (59–89 years old) BM-HLCs (Table 2). In contrast, only one gene RNF213 encoding ring finger protein 213 was reduced (*p* = 0.0411) in older donor BM-MSCs. Notably, the expression of TMEM173 encoding STING, an adaptor molecule mediating the cell’s responses to cytosolic DNA [47], as well as MTCH2, were significantly reduced in older donor BM-HLCs (*p* = 0.0152) (Table 2), which could, in theory, influence their cytoplasmic nucleic acid sensing and intracellular ROS accumulation, respectively. The expression of KL encoding an antiaging protein Klotho and IFN1 receptor IFNAR1 were also >2-fold reduced in old-donor BM-HLCs, albeit not significantly. As expected, age-related changes in BM-MSCs or BM-HLCs were lower in magnitude (maximum 5-fold for IL7R in HLCs) compared to cell type-specific differences between BM-MSCs and BM-HLCs where over 50-fold differences were commonly found (Table 1). Individual’s biological age does not always perfectly match their chronological age [48]. For this reason, the expression of two antiaging molecules KL and SIRT6 was next correlated with a number of ISGs in both CD4 ^low^CD271^+^ BM-MSCs and CD45^+^CD271^−^ BM-HLCs (Figure 5). Significant, strong positive correlations between KL and RTP4 and KL and SIRT6 were found in both BM-MSCs (*r* = 0.9455, *p* = 0.001 for RTP4 and *r* = 0.7182, *p* = 0.02 for SIRT) and BM-HLCs (*r* = 0.8364, *p* = 0.005 for RTP4 and *r* = 0.8, *p* = 0.005 for SIRT6), but only in BM-MSCs did KL additionally correlate with IRF3 (*r* = 0.9, *p* = 0.001), OAS3 (*r* = 0.7545, *p* = 0.02), CCND2 (*r* = 0.8909, *p* = 0.001) and BST2 (*r* = 0.8626, *p* = 0.002) (Figure 5a). A similar pattern was found for SIRT6 although a number of coregulated genes was higher and included IFNAR2, IRF9, IFI35, RNF213, RTP4 and KL; nevertheless, only in BM-MSCs did SIRT6 additionally correlate with BST2 (*r* = 0.8636, *p* = 0.002), OAS3 (*r* = 0.8273, *p* = 0.002), IRF3 (r = 0.8364, *p* = 0.005) and CCND2 (*r* = 0.7182, *p* = 0.02) (Figure 5b). In addition to our data showing an age-related TMEM173/STING downregulation in BM-HLCs, but not in BM-MSCs, these data pointed towards a potentially heightened state of readiness for DDR-related nucleic acid sensing and combating senescence in BM-MSCs compared to BM-HLCs. To explore this possibility, we measured intracellular ROS levels in CD45^low^CD271^+^ BM-MSCs from 13 donors (aged 25–89 years old). While no notable increases in basal ROS in BM-MSCs from older-aged donors were found (Figure 5c), interesting trends for positive correlations were observed between basal ROS and the expression levels of both TMEM173/STING and MTCH2 in BM-MSCs (Figure 5d), providing initial support for this idea.

### 3.5. Differences in IFN1 Profile of CD45^low^CD271^+^ BM-MSCs and CD45^+^CD271^−^ BM-HLCs in Patients with Osteoarthritis (OA)

**Figure 6:**
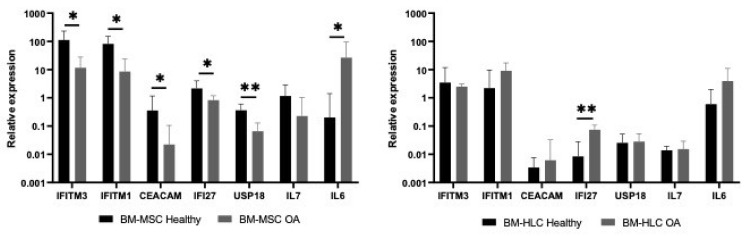
Relative expression of selected IFN signature genes in both cell types (left panel: BM-MSCs, right panel: BM-HLCs) in healthy old donors (*n* = 6) and age-matched OA patients (*n* = 7). Data is represented as medians and error bars indicate interquartile rages. * *p* < 0.05 and ** *p* < 0.01, Mann–Whitney U-test. All genes except IL7 present statistically significant reduction in BM-MSCs from osteoarthritis (OA) patients compared to healthy old donors. BM-HLCs present a different pattern, with IFI27 being significantly upregulated in OA.

To investigate how IFN1 gene expression in BM-MSCs may change in a pathological condition, we next compared the IFN1 signature in native CD45^low^CD271^+^ BM-MSCs from healthy older subjects (age range 59–89 years old) with age-matched patients with osteoarthritis (OA) (age range 56–82 years old). To explore cell-specificity of these changes, the IFN1 signature in CD45^+^CD271^−^ BM-HLCs was also compared (Figure 6).

As seen in this figure, prominent, statistically significant downregulation in several ISGs was observed in BM-MSCs, which was clearly BM-MSC-specific as similar-level changes were not evident in BM-HLCs. IL7 expression was reduced (albeit nonsignificantly) in OA BM-MSCs in parallel to IFITM1 and IFITM3 (Figure 6), consistent with data on the correlation between these transcripts in healthy BM-MSCs (Figure 4b). On the other hand, IL6 expression was induced in both OA BM-MSCs and BM-HLCs, consistent with previous studies on the overall increase in IL6 production in OA [49]. The full list of genes differentially expressed between OA BM-MSCs and OA BM-HLCs is provided in Table 3.

## 4. Discussion

This study investigated the IFN1 gene expression profile of human BM-MSCs in their native state and upon culture and IFN1 stimulation. For the first time, it showed the expression of IFN1 receptors and many ISGs in native uncultured human BM-MSCs, as well as dramatic changes in BM-MSC IFN1 receptor expression following standard culture and IFNα and IFNβ stimulation. This indicated an ability of BM-MSCs to actively regulate their IFN1 receptor expression and autocrine IFN production in response to exogenous IFN1s.

Previous pioneering work by James et al. has proposed the existence of two subpopulations of BM-MSCs different in their IFN1 profiles as well as their differentiation, migratory and immunoregulatory capacities (exemplified by clonal lines Y201 and Y202) [20]. However, these immortalised cell lines were produced by virus-mediated telomerase gene transfer into the cells, therefore a possibility of induced anti-viral IFN1 response in these BM-MSCs could not be excluded. The current investigation has shown constitutive IFN1 signalling in uncultured and nonvirally-transduced BM-MSCs from healthy donors, therefore revealing its homeostatic role in health. It also independently confirmed a higher-level IFN1 pathway activity in Y202 cells compared to Y201 cells, and, significantly, revealed a larger similarity of native BM-MSCs to Y201 cells rather than Y202 cells. Such IFN1 gene expression profile indicated a moderate level of IFN1 pathway activity in native human BM-MSCs.

In this study we compared native, uncultured BM-MSCs with a broad population of hematopoietic lineage cells (BM-HLCs), similar to our previous publications [15,24]. We observed clear differences in IFN1 profiles between BM-MSCs and BM-HLCs despite their residence in the same tissue environment. Firstly, BM-MSCs expressed higher levels of IFN1 receptors, which might translate into their higher responsiveness to minute amounts of IFN1s. Secondly, 17-fold higher expression of USP18 in BM-MSCs than in BM-HLCs could indicate higher MSC responsiveness to IFNβ. Τhis molecule has been recently proposed to be a negative regulator of IFN1 signalling affecting cell viability and response to viral infection at multiple levels [50,51], and “a prime candidate to explain differential activation via IFNβ versus IFNα” [52]. The authors have also proposed that cell-specific, “tunable” IFN1 activities, such antiproliferative and immunomodulatory activities, require high-affinity IFNβ binding and high receptor densities. In the current study, we have demonstrated stronger antiproliferative effects of IFNβ compared to IFNα on human BM-MSCs, in addition to showing their higher IFN1 receptor transcript levels compared to BM-HLCs. This points towards a potentially prominent role of IFNβ signalling in BM-MSC homeostasis. In agreement, another study has documented an important role of IFNβ in the induction of BM-HSC niche-supporting phenotype in the BM-MSCs and a parallel inhibition of their osteoblast differentiation [53].

As IFN1 responses have been also linked to stem cell aging and senescence, we explored any differences in IFN1 profiles in native BM-MSCs and BM-HLCs in relation to donor age. While in BM-HLCs, several IFN1 ISGs were reduced in older donors, this was not observed in BM-MSCs. In fact, while an antiaging gene KL was reduced in BM-HLCs in direct correlation with several ISGs, this was not the case in BM-MSCs, in which KL correlated with a broader range of ISGs, suggesting a possibility for an internal feedback mechanism of its regulation. It could be speculated that the higher levels of STING in BM-MSCs reflect a potentially higher state of “readiness” for the recognition of DDR-based cytosolic nucleic acids in BM-MSCs in comparison to BM-HLCs [47] that, consequently, could explain no significant age-related differences in basal ROS intracellular levels in BM-MSCs. Our functional experiments showing trends for positive relationships between STING and MTCH2 gene expression and basal ROS accumulation in uncultured BM-MSCs are encouraging and should be validated in larger donor cohorts. These cytoplasmic nucleic acid sensing mechanisms may also facilitate BM-MSC longevity, stemness and quiescence [17], which would be valuable to explore in the future. Interestingly, the expression of two ISGs (SERPING1 and OAS2) has been recently shown to be better maintained in three-dimensional (3D) BM-MSC culture compared to their 2D culture [25]. Three-dimensional culture much better preserves the BM-MSC self-renewal ability, the prime characteristic of “stemness” in different types of stem cells [54], suggesting that SERPING1, also found to be highly BM-MSC-specific in the present study, could be an interesting candidate for the study of BM-MSC self-renewal in vivo.

IFN1-tunable immunomodulatory function in vivo can be related to their control of osteoclast formation [55,56] as well as lymphocyte development, maturation and memory cell retention [41], with both processes known to be strongly affected by age. In the present study, we observed a strong positive correlation between BM-MSC IL7 transcript expression, involved in both lymphocyte development and memory cells retention in the BM, and the expression of several ISGs. IL7 transcription in BM-MSCs can be controlled by regulatory factors activated by lymphocyte adhesion or extrinsic IFNβ stimulation, and subsequently contribute to the myeloid skewing and inflamm-aging processes in the elderly, as well as the progression of OA [57]. IL7 transcript showed a trend for downregulation in BM-MSCs from OA patients in parallel with significant downregulation of several ISGs including IFITM1, IFITM3 and USP18; this pointed towards potential involvement of IFN1 signalling pathway in IL7 transcriptional regulation in OA. Remarkably, the expression of RANKL in BM-MSCs, a factor inducing osteoclast differentiation and enhancing bone resorption [58], negatively correlated with many analysed ISGs in the current study. This potentially implied a protective role of IFN1 signalling in the BM-MSC control of bone resorption, which merits further investigations. Although we did not observe particular correlations between ISG expression and classical bone formation transcripts in native BM-MSCs, functional studies indicating a decline in osteoblasts [32] and the impairment in extracellular matrix formation and mineralisation by IFN1 [53] indicate that further investigations are needed to better understand the role of constitutive IFN1 signaling in the maintenance of bone strength.

This study is limited by low numbers of donors in younger and older age groups. However, we uniquely used healthy BM donors and purified rare BM-MSCs using FACS sorting. Additionally, a broader panel of genes, including genes involved in autophagy, as well as more ISGs and antiaging genes, is needed to unravel the involvement of IFN1 signalling in BM-MSC survival and aging processes. We were limited by the well-known low frequency of native BM-MSCs in BM aspirates [13,15], as well as the volumes of aspirates that could be ethically sourced from human participants. This precluded direct evaluation of the effects of IFN1 stimulation on uncultured BM-MSCs, which is additionally compounded by low clonogenicity of sorted BM-MSCs [59], possibly due to cell stress induced by cell sorting [60]. It is also unclear whether there is cell-to-cell heterogeneity within the sorted fraction of native BM-MSCs. Indeed, different subpopulations of native BM-MSCs have been previously described [61,62], some of which may indeed differ in their IFN1 signatures [20]. Novel methods such as single-cell RNA sequencing [63] on larger cohorts of donors will be the next step forward for addressing these questions. Furthermore, a proteomic analysis such as Western blot, enzyme-linked immunosorbent assay (ELISA) or flow cytometric evaluation of critically important IFN1 genes in BM-MSCs would be required to have a greater view regarding the impact of IFNα and IFNβ in BM-MSCs biology.

In summary, IFN1 responses in cells have been initially related to their antiviral activity, but more recently, they have been linked to a broader range of “tonic” activities, including the control of cell homeostasis, proliferation, immunomodulation, quiescence and senescence [45,64,65]. In this study, we presented the homeostatic IFN1 profile of native human BM-MSCs and showed its difference to hematopoietic, immune-lineage cells from the same BM microenvironment. Considering a large proportion of selected IFN1 genes were overexpressed in BM-MSCs in comparison to BM-HLCs and, in contrast, fewer age-related differences were found in BM-MSCs, BM-MSCs in vivo may uniquely utilise IFN1 signalling machinery to resist aging influences compared to BM-resident immune-lineage cells. This machinery may allow them to tolerate endogenous as well as exogenous ROS and, in turn, help to prevent BM-HSC senescence [66]. The current work therefore opens new avenues for a broader exploration of IFN1 signalling in native BM-MSCs towards its potential modulation for the prevention of age-related inflamm-aging and bone frailty in the elderly.

## Figures and Tables

**Table 1:** IFN1-stimulated genes (ISGs) and senescence-activated secretory phenotype (SASP)/senescence-related transcripts differentially-expressed between bone marrow multipotential stromal cells (BM-MSCs, CD45^low^CD271^+^ cells) and hematopoietic-lineage cells (BM-HLCs; CD45^+^CD271^−^ cells).

Gene Name	Gene Id	MSC (Median)	HLC (Median)	Fold Difference	*p* Value
SERPING	Serine protease inhibitor, clade G (C1 inhibitor), member 1	22	0.094	233.3	0.0003
IFI27	Interferon gamma-inducible protein 27	1.551	0.01	155.1	<0.0001
PRDM16	PR domain containing 16	0.179	0.002	105.3	0.042
IL7	Interleukin 7	1.481	0.016	93.7	0.0005
CEACAM	Carcinoembryonic antigen-related cell adhesion molecule 1	0.31	0.007	44.3	0.0021
IFITM3	Interferon induced transmembrane protein 3	137.9	3.79	36.4	<0.0001
KL	Klotho	0.51	0.02	25.5	0.0083
USP18	Ubiquitin specific peptidase 18	0.35	0.02	17.5	<0.0001
LRP1	LDL Receptor Related Protein 1	48.92	4.03	12.1	0.0007
RTP4	Receptor (chemosensory) transporter protein 4	0.96	0.08	12	0.0036
IFITM1	Interferon induced transmembrane protein 1	75.05	6.33	11.9	0.0005
LY6E	Lymphocyte antigen 6 complex, locus E	24.58	2.4	10.2	<0.0001
SPATS2L	Spermatogenesis associated, serine-rich 2-like	3.509	0.405	8.7	0.0001
IFI6	Interferon inducible alpha protein 6	3.285	0.438	7.5	0.0145
IFIT5	Interferon induced protein with tetratricopeptide repeats 5	0.633	0.11	5.8	0.0284
CCND2	CyclinD2	2.359	0.415	5.7	0.0013
SOCS1	Suppressor of cytokine signalling 3	2.558	0.461	5.6	0.0003
ABCA1	ATP Binding Cassette Subfamily A Member 1	1.173	0.24	4.9	0.0249
IFNAR1	Interferon alpha receptor 1	4.28	0.88	4.9	0.0018
IFI44L	Interferon induced protein 44 like	1.507	0.31	4.9	0.008
OASL	2′-5′-oligoadenylate synthetase	1.014	0.22	4.6	0.0284
PLSCR1	Phospholipid scramblase 1	4.117	0.998	4.1	0.0068
EIF2AK2	Eukaryotic translation initiation factor 2-alpha kinase 2	3.807	0.956	4	0.0023
OAS3	2′-5′-oligoadenylate synthetase 3	2.55	0.7	3.6	0.0121
IFI35	Interferon inducible alpha protein 35	2.68	0.76	3.5	0.0387
NT5C3B	5′-nucleotidase cytosolic IIIB	0.56	0.159	3.5	0.0018
RSAD2	Radical S-adenosyl methionine domain containing 2	0.434	0.132	3.3	0.0169
IFNAR2	Interferon alpha receptor 2	5.82	1.84	3.2	0.0332
IFI44	Interferon inducible alpha protein 44	3.1	1.01	3.1	0.0205
EPSTI1	Epithelial stromal interaction 1	1.59	0.57	2.8	0.0045
IFIH1	Interferon induced with helicase C domain 1	0.45	0.17	2.7	0.0106
UBE2L6	Ubiquitin/ISG15-conjugating enzyme E2 L6	11.86	4.5	2.6	0.0083
SAMD9L	Sterile alpha motif domain containing 9-like	1.67	0.642	2.6	0.0387
BST2	Bone marrow stromal antigen 2, Tetherin (CD317)	12.06	4.97	2.4	0.0083
IFI16	Interferon inducible alpha protein 16	1.664	0.809	2.1	0.0068
RGS1	Regulator of G protein signalling 1	0.621	32.29	52	0.0184
IL8	Interleukin 8	2.919	67.21	23	0.0022
TNF	Tumour necrosis factor	0.17	3.67	21.6	0.013
IRF5	Interferon regulatory factor 5	0.129	1.285	10	0.0157
HPSE	Heparanase	0.097	0.732	7.6	0.0171
FCGR1B	Fc fragment of IgG receptor 1B	0.19	1.04	5.5	0.0031
BID	BH3 interacting domain	0.21	1	4.8	0.0188
PRDM1	PR domain containing 1	0.51	1.72	3.4	0.0148
LAIR1	Leukocyte associated immunoglobulin like receptor1	0.43	1.4	3.3	0.0278
CASP1	Caspase1	3.06	6.16	2	0.0205

Gene expression was normalised to housekeeping gene HPRT1. The upper section of the table includes genes that were expressed significantly higher in MSCs than in HLCs, and the lower section of the table includes genes that were expressed significantly higher in HLCs than in MSCs.

**Table 2:** Age-related increase in expression of ISGs in BM-HLCs (CD45^+^CD271^−^ cells).

Gene ID	Gene Name	Young (Median)	Old (Median)	Fold Difference	*p* Value
IL7R	Interleukin7 receptor	0.491	0.098	5.01	0.026
PRKRA	Protein Kinase, Interferon-Inducible Double Stranded RNA Dependent Activator	0.970	0.200	4.85	0.0152
SCARB1	Scavenger Receptor Class B Member 1	0.431	0.131	3.29	0.0087
CXCL10	C-X-C motif chemokine 10	0.580	0.180	3.22	0.0667
RTP4	Receptor transporter protein 4	0.150	0.050	3.00	0.0152
CEACAM	Carcinoembryonic antigen-related cell adhesion molecule 1	0.008	0.003	2.67	0.0667
Tp53	Tumour protein 53	2.930	1.210	2.42	0.0043
IRF9	Interferon regulatory factor 9	1.520	0.628	2.42	0.026
IRF3	Interferon regulatory factor 3	1.561	0.660	2.37	0.0411
ABCG1	ATP Binding Cassette Subfamily G Member 1	0.070	0.030	2.33	0.0649
TMEM173/STING	Transmembrane protein 173/Stimulator of interferon genes	2.293	1.033	2.22	0.026
IFIT5	Interferon induced protein with tetratricopeptide repeats 5	0.124	0.058	2.14	0.0411
MTCH2	Mitochondrial carrier homolog 2	1.610	0.790	2.04	0.0152

Gene expression was normalised to housekeeping gene HPRT1. Table includes genes that were expressed significantly higher in BM-HLCs form older donors, indicating an age-related increase in these ISGs in vivo.

**Table 3:** Differentially expressed IRGs in OA.

Gene ID	Gene Name	MSC (Median)	HLC (Median)	Fold Difference	*p* Value
**PRDM16**	PR domain containing 16	0.11	0.001	110	0.055
**SERPING**	Serine protease inhibitor, clade G (C1 inhibitor), member 1	8.8	0.086	102.3	0.0006
**Kl**	Klotho	0.1	0.002	50	0.0175
**LRP1**	LDL Receptor Related Protein 1	8	0.31	25.8	0.0111
**IL7**	Interleukin 7	0.22	0.015	14.7	0.0012
**IFI27**	Interferon gamma-inducible protein 27	0.82	0.07	11.7	0.0006
**OAS1**	2′-5′-oligoadenylate synthetase 1	0.23	0.024	9.6	0.022
**CHMP5**	Charged multivesicular body protein 5	1.78	0.21	8.5	0.0728
**IL6**	Interleukin 6	26.57	3.93	6.8	0.0728
**SPATS2L**	Spermatogenesis associated, serine-rich 2-like	2.12	0.317	6.7	0.0012
**TLR4**	Toll Like Receptor 4	0.88	0.16	5.5	0.011
**NT5C3B**	5′-nucleotidase cytosolic IIIB	0.37	0.07	5.3	0.0041
**CCL8**	Chemokine C-C motif ligand 8	0.12	0.023	5.2	0.0006
**CXCL10**	C-X-C motif chemokine 10	0.56	0.11	5.1	0.038
**ABCA1**	ATP Binding Cassette Subfamily A Member 1	2.88	0.59	4.9	0.0728
**IFITM3**	Interferon induced transmembrane protein 3	11.6	2.47	4.7	0.026
**IFI35**	Interferon alpha-inducible protein 35	1.29	0.28	4.6	0.0728
**RTP4**	Receptor (chemosensory) transporter protein 4	0.12	0.03	4	0.0728
**PRKRA**	Protein Kinase, Interferon-Inducible Double Stranded RNA Dependent Activator	0.83	0.21	4.0	0.073
**GBP1**	Guanylate binding protein 1	3.3	0.84	3.9	0.038
**STING**	Stimulator of interferon genes	1.25	0.46	2.7	0.073
**PLSCR1**	Phospholipid scramblase 1	2.04	0.81	2.5	0.0262
**IFI44L**	Interferon induced protein 44 like	0.35	0.16	2.2	0.0262
**IFIT5**	Interferon induced protein with tetratricopeptide repeats 5	0.26	0.12	2.2	0.0006
**IFI16**	Interferon inducible alpha protein 16	1.164	0.56	2.1	0.0041
**IFNAR2**	Interferon alpha receptor 2	1.36	0.66	2.1	0.035
**IFNG**	Interferon gamma	0.003	1.29	430	0.0167
**RGS1**	Regulator of G protein signalling 1	0.18	21.98	122.1	0.0006
**LAMP3**	Lysosome associated membrane glycoprotein 3	0.02	0.48	24	0.0023
**FCGR1B**	Fc fragment of IgG receptor IB	0.003	0.065	21.7	0.0513
**IL7R**	Interleukin 7 receptor	0.19	2.89	15.2	0.0012
**SIGLEC1**	Sialic acid binding Ig like Lectin 1	0.008	0.08	10	0.0714
**PRDM1**	PR domain containing 1, with ZNF domain	0.71	3.6	5.1	0.011
**LAIR1**	Leukocyte associated immunoglobulin like receptor1	0.13	0.61	4.7	0.0023
**TGFB**	PR domain containing 1, with ZNF domain	2.9	13.23	4.6	0.0012
**ISG20**	Interferon stimulated exonuclease gene 20kDa	1.17	5.081	4.3	0.0006
**HPSE**	Heparanase	0.06	0.16	2.7	0.053

Top panel (PRDM10 to IFNAR2) includes ISGs that were expressed higher in MSCs over HLCs and the bottom panel (IFNG to HPSE) includes the IRGs expressed higher in HLCs over MSCs in OA.

## Supplementary Materials

Supplementary materials can be found at https://www.mdpi.com/2227-9059/8/7/214/s1.

## Abbreviations

IFN1	Type 1 interferon
IFNα	Interferon alpha
IFNβ	Interferon beta
MSCs	Mesenchymal stromal cells
BM	Bone marrow
HSCs	Hematopoietic stem cells
KL	Klotho
SIRT6	Sirtuin 6
ISGs	Interferon stimulated genes
DNA	Deoxyribonucleic acid
SSEA4	Stage specific embryonic antigen 4
cGAS/STING	Cyclic GMP-AMP synthase/Stimulator of interferon genes
IFNAR1	Interferon alpha receptor 1
IFNAR2	Interferon alpha receptor 2
JAK	Janus kinase
STAT1	Signal transducer and activator of transcription1
Mx1	MX dynamine like GTPase 1
HERC5	HECT and RLD domain containing E3 ubiquitin protein ligase 5
IFTM1	Interferon induced trans membrane protein 1
IFITM3	Interferon induced trans membrane protein 3
IRF7	Interferon regulatory factor 7
RPT4	Receptor transporter protein 4
ISG20	Inteferon stimulated gene 20
BID	BH3 interacting domain
MTCH2	Mitochondrial homologue carrier 2
ROS	Reactive oxygen species
OA	Osteoarthritis
OAS3	2′-5′-oligoadenylate synthetase 3
SASP	Senescence associated secretory phenotype
IL6	Interleukin 6
IL8	Interleukin 8
USP18	Ubiquitin specific peptidase 18
FABP4	Fatty acid binding protein 4
PPARγ	Peroxisome proliferator activator receptor gamma
RUNX2	Runt related transcription factor 2
SPARC	Secreted protein acid and cysteine rich/Osteonectic
SOX9	SRY-Box Transcription Factor 9
ACAN	Aggrecan
IL7	Interleukin 7
CXCL12	C-X-C chemokine 12
TNFSF11	Tumour necrosis factor super family 11
Ly6E	Lymphocyte antigen 6E
EIF2AK2	Eukaryotic translation initiation factor 2-alpha kinase 2
TMEM173	Transmembrane protein 173/STING
IRF3	Interferon regulatory factor 3
CCND2	CyclinD2
IRF9	Interferon regulatory factor 9
IFI35	Interferon induced 35
RNF213	Ring finger protein 213
DDR	DNA-damage response
3D	Three dimensional
SERPING1	Serpin family G member1
EDTA	Ethylene diamine tetra acetic acid
BMA	Bone marrow aspirate
DMSO	Dimethylsulfoxide
qPCR	quantitative Polymerised chain reaction
IFC	Integrated fluid circuits
DPBS	Dulbeccos phosphate buffer saline
