# Supplementary material for: Intrinsic Type 1 Interferon (IFN1) Profile of Uncultured Human Bone Marrow CD45lowCD271+ Multipotential Stromal Cells (BM-MSCs): The Impact of Donor Age, Culture Expansion and IFNα and IFNβ Stimulation
Source: Biomedicines. 2020 Jul 15;8(7):214. doi: 10.3390/biomedicines8070214 (PMC7399891; doi:10.3390/biomedicines8070214)
Supplement: Supplementary file 1 [file biomedicines-08-00214-s001.pdf]

Supplementary data

Supplementary table 1: List of 102 genes investigated in this study and their assay ID

Gene	Full name	Assay
TNFRSF11B/OPG	Osteoprotegerin	Hs00900360_m1
SFRP1	Secreted frizzled related protein 1	Hs00610060_m1
CXCL12	C-X-C motif chemokine 12	Hs00171022_m1
FABP4	Fatty acid binding protein 4	Hs00609791_m1
LepR	Leptin Receptor, encoding CD295 protein	Hs00174492_m1
SPARC	Secreted protein acidic and rich in cysteine, Osteonectin	Hs00277762_m1
TNFSF11/RANKL	Receptor activator of nuclear factor kappa-B Ligand	Hs01092186_m1
PPAR-ɣ	Peroxisome proliferator activated receptor - gamma	Hs01115513_m1
GJA1	Gap Junction alpha 1, encoding Cx43 protein	Hs00748445_s1
RUNX2	Runt related transcription factor 2	Hs00231692_m1
IFNA1	Interferon alpha 1	Hs00855471_g1
IFNB1	Interferon beta 1	Hs01077958_s1
IFNAR1	Interferon alpha receptor 1	Hs01066118_m1
IFNAR2	Interferon alpha receptor 2	Hs00174198_m1
STING	Stimulator of interferon genes	Hs00736956_m1
IRF3	Interferon regulatory factor 3	Hs01547282_m1
STAT1	Signal transducer and activator or transcription 1	Hs01013996_m1
BID	BH3 interacting domain	Hs00609632_m1
MTCH2	Mitochondrial carrier homolog 2	Hs00819318_g1
Kl	Klotho	Hs00183100_m1
Sirt6	Sirtuin 6	Hs00966002_m1
Tp53	Tumour protein 53	Hs01034249_m1
IL6	Interleukin 6	Hs00985639_m1
IL7	Interleukin 7	Hs99999033_m1

IL8	Interleukin 8	Hs00174103_m1
ABCA1	ATP Binding Cassette Subfamily A Member 1	Hs00194045_m1
ABCG1	ATP Binding Cassette Subfamily G Member 1	Hs00245154_m1
AICDA	activation-induced cytidine deaminase	Hs00757808_m1
BST2	Bone marrow stromal antigen 2, Tetherin (CD317)	Hs01561315_m1
CASP1	Caspase1	Hs00354836_m1
CCL8	Chemokine C-C motif ligand 8	Hs04187715_m1
CCND2	CyclinD2	Hs00153380_m1
CEACAM	Carcinoembryonic antigen-related cell adhesion molecule 1	Hs00989786_m1
CHMP5	charged multivesicular body protein 5	Hs00603789_mH
CXCL10	C-X-C motif chemokine 10	Hs01124251_g1
EPSTI1	Epithelial stromal interaction 1 (breast)	Hs01566789_m1
EIF2AK2	Eukaryotic translation initiation factor 2-alpha kinase 2	Hs00169345_m1
FCGR1B	Fc fragment of IgG receptor Ib	Hs00174081_m1
GBP1	Guanylate binding protein 1	Hs00977005_m1
GUSB	Glucuronidase beta	Hs99999908_m1
HERC5	Hect domain and RLD 5	Hs00180943_m1
HPSE	Heparanase	Hs00935036_m1
IFI6	Interferon alpha-inducible protein 6	Hs00242571_m1
IFI16	Interferon alpha-inducible protein 16	Hs00194261_m1
IFI27	Interferon alpha-inducible protein 27	Hs01086373_g1
IFI35	Interferon alpha-inducible protein 35	Hs00413458_m1
IFI44	Interferon alpha-inducible protein 44	Hs00951349_m1
IFI44L	Interferon alpha-inducible protein 44-like	Hs00915292_m1
IFIH1	Interferon induced with helicase C domain 1	Hs01070332_m1
IFIT1	Interferon-induced protein with tetratricopeptide repeats 1	Hs01911452_s1
IFIT2	Interferon-induced protein with tetratricopeptide repeats 2	Hs00533665_m1
IFIT3	Interferon-induced protein with tetratricopeptide repeats 3	Hs01922752_s1
IFIT5	Interferon-induced protein with tetratricopeptide repeats 5	Hs00202721_m1

IFITM1	Interferon induced transmembrane protein 1	Hs00705137_s1
IFITM3	Interferon induced transmembrane protein 3	Hs03057129_s1
IFNG	Interferon gamma	Hs00989291_m1
IL7R	Interleukin 7 receptor	Hs00904815_m1
IRF2	Interferon regulatory factor 2	Hs01082884_m1
IRF5	Interferon regulatory factor 5	Hs00158114_m1
IRF7	Interferon regulatory factor 7	Hs01014809_g1
IRF9	Interferon regulatory factor 9	Hs00196051_m1
ISG15	Interferon stimulated exonuclease gene 15kDa	Hs00192713_m1
ISG20	Interferon stimulated exonuclease gene 20kDa	Hs00158122_m1
LAIR1	Leukocyte associated immunoglobulin like receptor 1	Hs01083919_g1
LAMP3	Lyosome associated membrane glycoprotein 3	Hs00180880_m1
LRP1	Low density lipoprotein receptor-related protein 1	Hs01059275_m1
LY6E	Lymphocyte antigen 6 family member E	Hs03045111_g1
MSR1	Macrophage Scavenger Receptor 1	Hs00234007_m1
MX1	Myxovirus resistance 1, interferon-inducible protein p78	Hs00895608_m1
NT5C3B	5'-nucleotidase cytosolic IIIB	Hs00369454_m1
OAS1	2'-5'-oligoadenylate synthetase 1	Hs00973640_m1
OAS2	2'-5'-oligoadenylate synthetase 2	Hs00942650_m1
OAS3	2'-5'-oligoadenylate synthetase 3	Hs00196324_m1
OASL	2'-5'-oligoadenylate synthetase-like	Hs00984387_m1
PHF11	PHD finger protein 11	Hs00211573_m1
PPIA	Peptidylpropyl isomerase A	Hs99999904_m1
PRDM1	PR domain containing 1, with ZNF domain	Hs00153357_m1
PRDM16	PR domain containing 16, with ZNF domain	Hs00223161_m1
PRKRA	Protein Kinase, Interferon-Inducible Double Stranded RNA Dependent Activator	Hs00269379_m1
RGS1	Regulator of G-protein signaling 1	Hs01023772_m1
RNF213	Ringer finger protein 213	Hs00326306_m1
RSAD2	Radical S-adenosyl methionine domain containing 2	Hs00369813_m1

RTP4	Receptor (chemosensory) transporter protein 4	Hs00223342_m1
SAMD9L	Sterile alpha motif domain containing 9-like	Hs00416109_m1
SCARB1	Scavenger Receptor Class B Member 1	Hs00969826_m1
SERPING	Serpin peptidase inhibitor, clade G (C1 inhibitor), member 1	Hs00163781_m1
SIGLEC1	Sialic acid binding Ig like Lectin 1	Hs00988063_m1
SOCS1	PR domain containing 1; with ZNF domain;hCG33515 Celera Annotation	Hs00705164_s1
SP100	RAR-related orphan receptor C;hCG16918 Celera Annotation	Hs00162109_m1
SPATS2L	Spermatogenesis associated, serine-rich 2-like	Hs01016364_m1
TAP1	Transporter 1, ATP Binding Cassette Subfamily B Member	Hs00388675_m1
TGFB	Transforming Growth Factor Beta 1	Hs00998133_m1
TLR4	Toll Like Receptor 4	Hs00152939_m1
TNF	Tumour necrosis factor	Hs00174128_m1
TNFRSF11A/RANK	tumor necrosis factor receptor superfamily member 11a	Hs00921372_m1
TRIM38	Tripartite motif containing 38	Hs00197164_m1
UBE2L6	Ubiquitin/ISG15-conjugating enzyme E2 L6	Hs01125548_m1
UNC93B	Unc-93 Homolog B1, TLR Signaling Regulator	Hs00276771_m1
USP18	Ubiquitin specific peptidase 18	Hs00276441_m1
XAF1	XIAP associated factor 1	Hs01550142_m1
HPRT1	Hypoxanthine phosphoribosyl transferase (housekeeping)	Hs99999909_m1

Supplementary table 2: List of genes used in the investigation of IFN α and β stimulations and their assay ID

Gene	Full name	Assay
ADIPOQ	Adiponectin	Hs00605917_m1
FABP4	Fatty acid binding protein 4	Hs00609791_m1
PPARγ	Peroxisome proliferator-activated receptor gamma	Hs01115513_m1
ACN	Aggrecan	Hs00153936_m1
COL2A1	Type II collagen	Hs00264051_m1
SOX9	Sex determining region Y box 9	Hs00165814_m1
ALP	Alkaline phosphatase	Hs00758162_m1
BGLAP	Bone gamma-carboxyglutamate protein	Hs01587814_g1
RUNX2	Runt-related transcription factor 2	Hs00231692_m1
COL1A1	Type I collagen	Hs1076777_m1
HPRT	Hypoxanthine-guanine phosphoribosyltransferase	Hs99999905_m1
PPIA	Peptidylprolyl isomerase A	Hs99999904_m1
IFNA	Interferon alpha	Hs00855471_g1
IFNB	Interferon beta	Hs01077958_s1
IFNAR1	Interferon alpha/beta receptor 1	Hs01066118_m1
IFNAR2	Interferon alpha/beta receptor 2	Hs00174198_m1
BST2	Bone marrow stromal cell antigen 2	Hs01561315_m1
EIF2AK2	Eukaryotic translation initiating factor 2 alpha kinase 2	Hs00169345_m1
HERC5	HECT and RLD domain containing E3 ubiquitin protein ligase 5	Hs00180943_m1
IFI27	Interferon inducible protein 27	Hs01086373_g1
IFI44	Interferon induced protein 44	Hs00951349_m1
IFI44L	Interferon induced protein 44 like	Hs00915292_m1
IFIH1	Interferon induced with helicase C domain 1	Hs01070332_m1
IFIT3	Interferon induced protein with tetratricopeptide repeats	Hs01922752_s1
IFITM1	Interferon induced transmembrane protein 1	Hs00705137_s1

IRF7	Interferon regulatory factor 7	Hs01014809_g1
ISG20	Interferon stimulated exonuclease gene 20	Hs00158122_m1
MX1	MX dynamin like GTPase 1	Hs00895608_m1
OAS3	2’-5’oligoadenylate synthetase 3	Hs00196324_m1
RSAD2	Radical S-adenosyl methionine domain containing 2	Hs00369813_m1
RPT4	Receptor transporter protein 4	Hs00223342_m1
STAT1	Signal transducer and activator of transcription 1	Hs01013996_m1
USP18	Ubiquitin specific peptidase 18	Hs00276441_m1
RNF213	Ring finger protein 213	Hs00326306_m1
BID	BH3 interacting-domain death	Hs00609632_m1
MTCH2	Mitochondrial carrier 2	Hs00819318_g1
PRDM1	PR domain containing 1	Hs00153357_m1
PRDM16	PR domain containing 16	Hs00223161_m1
KL	Klotho	Hs00183199_m1
SIRT6	Sirtuin 6	Hs00966002_m1
p53	Tumour protein 53	Hs01034249_m1
IL6	Interleukin 6	Hs00985639_m1
IL8	Interleukin 8	Hs99999035_m1
CXCL1	C-X-C motif chemokine ligand 1	Hs00236937_m1
CCL2	C-C motif chemokine ligand 2	Hs00234140_m1
CCL8	C-C motif chemokine ligand 2	Hs04187715_m1
CDKN1A	Cyclin dependent kinase inhibitor 1A (AKA p21)	Hs00355782_m1
CDKN2A	Cyclin dependent kinase inhibitor 2A (AKA p16)	Hs00923894_m1

Supplementary figure 1

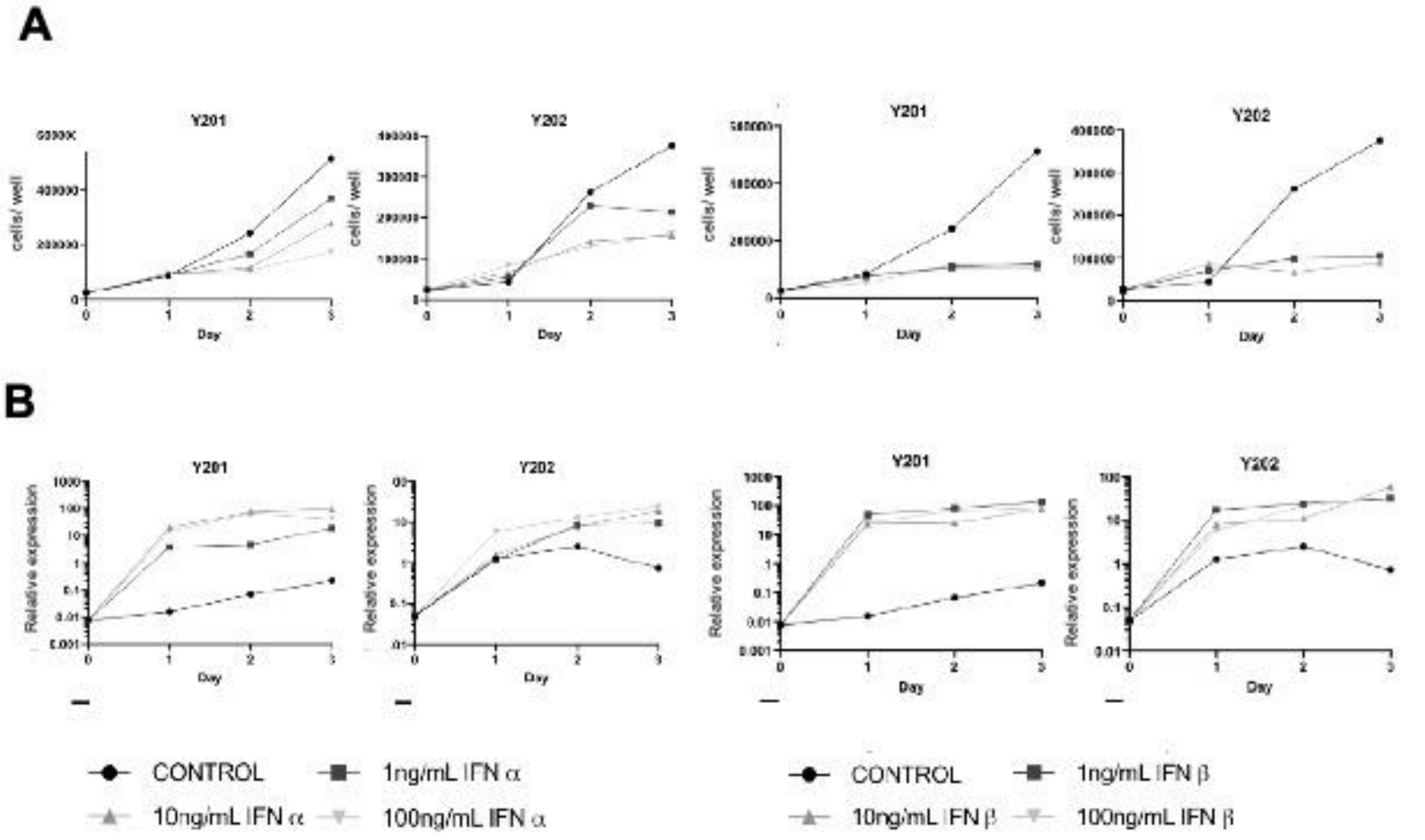

Supplementary figure 1: Time course and dose-response of cell proliferation (A) and BST2 expression (B) in clonal Y201 and Y202 BM-MSC cell lines. Left panels – treated with IFNα, right panels – treated with IFNβ.
